# Intricate subcellular journey of nanoparticles to the enigmatic domains of endoplasmic reticulum

**DOI:** 10.1080/10717544.2023.2284684

**Published:** 2023-11-21

**Authors:** Koyeli Girigoswami, Pragya Pallavi, Agnishwar Girigoswami

**Affiliations:** Medical Bionanotechnology, Faculty of Allied Health Sciences, Chettinad Hospital & Research Institute (CHRI), Chettinad Academy of Research and Education (CARE), Kelambakkam, Chennai, TN, India

**Keywords:** ER stress, nanoformulations, cancer, neurodegenerative disorder, hepatic disorder, drug delivery

## Abstract

It is evident that site-specific systemic drug delivery can reduce side effects, systemic toxicity, and minimal dosage requirements predominantly by delivering drugs to particular pathological sites, cells, and even subcellular structures. The endoplasmic reticulum (ER) and associated cell organelles play a vital role in several essential cellular functions and activities, such as the synthesis of lipids, steroids, membrane-associated proteins along with intracellular transport, signaling of Ca^2+^, and specific response to stress. Therefore, the dysfunction of ER is correlated with numerous diseases where cancer, neurodegenerative disorders, diabetes mellitus, hepatic disorder, etc., are very common. To achieve satisfactory therapeutic results in certain diseases, it is essential to engineer delivery systems that can effectively enter the cells and target ER. Nanoparticles are highly biocompatible, contain a variety of cargos or payloads, and can be modified in a pliable manner to achieve therapeutic effectiveness at the subcellular level when delivered to specific organelles. Passive targeting drug delivery vehicles, or active targeting drug delivery systems, reduce the nonselective accumulation of drugs while reducing side effects by modifying them with small molecular compounds, antibodies, polypeptides, or isolated bio-membranes. The targeting of ER and closely associated organelles in cells using nanoparticles, however, is still unsymmetrically understood. Therefore, here we summarized the pathophysiological prospect of ER stress, involvement of ER and mitochondrial response, disease related to ER dysfunctions, essential therapeutics, and nanoenabled modulation of their delivery to optimize therapy.

## 1. Introduction

The concept of delivering drugs directly to the intended site of action presents a significant opportunity to enhance the effectiveness of treating various diseases and disorders (Senapati et al., 2018). However, indiscriminate drug accumulation poses significant challenges, including inadequate bioavailability of therapeutic agents when administered using conventional dosing methods and the risk of off-target toxicity. These limitations greatly restrict the clinical utility of certain drugs. Fortunately, advancements in molecular and cellular biology have deepened our knowledge of subcellular structures and functions. Additionally, progress in pharmaceutics and nanotechnology has enabled the development of controlled drug release profiles, particularly in targeted drug delivery (Yang & Webster, 2009; Harini et al., 2022). This approach aims to minimize undesired side effects while maximizing therapeutic efficacy. By harnessing these scientific breakthroughs, we can overcome the drawbacks associated with nonspecific drug accumulation and improve the outcomes of treatment for various medical conditions.

The endoplasmic reticulum (ER) and mitochondria are essential organelles within cells that serve crucial functions in regulating cellular homeostasis and facilitating the turnover of biomolecules (Godoy et al., 2021). They are involved in numerous physiological processes as well as pathological activities that impact the survival of cells. Perturbations such as hypoxia, nutrient deprivation, oxidative stress, and microbial invasion can lead to a wide range of abnormalities in the ER and mitochondria (Wadgaonkar & Chen, 2021; Almanza et al., 2019). Consequently, these disruptions contribute to the development and progression of various diseases, including malignancies, neurodegenerative disorders, chronic liver disease, atherosclerosis, and ocular conditions (Ozcan & Tabas, 2012; da Silva et al., 2020). To address these diseases and disorders effectively, there is a growing interest in developing targeted drug delivery systems that preferentially deliver therapeutic molecules to the affected ER and mitochondria. This approach holds great promise in improving treatment outcomes by specifically addressing the molecular lesions associated with these subcellular organelles. By delivering drugs directly to the diseased sites, it is possible to enhance the efficacy of treatment while minimizing adverse effects, offering new avenues for managing these complex conditions.

Targeting the subcellular organelles of the ER and mitochondria necessitates the utilization of specific small molecular compounds and macromolecules possessing distinct structural characteristics (Yousif et al., 2009; Biasutto et al., 2019). To achieve site-specific accumulation, these compounds can either be directly coupled to therapeutic agents or attached to drug delivery systems (D'Souza & Weissig, 2009). However, it is crucial to acknowledge that direct conjugation might negatively impact the structure and pharmacological activities of drugs due to the utilization of denaturing solvents and toxic intermediates during organic synthesis. Alternatively, nanoparticle-based delivery platforms, particularly lipid nanoparticles, present a promising strategy (Yoo et al., 2022; Banerjee et al., 2017). These nanoparticles possess moderate drug encapsulation capabilities without the need for personalized synthesis, exhibit high biosafety, can accommodate diverse cargo, and offer tunable physicochemical properties. Consequently, lipid nanoparticles have emerged as potential candidates for clinical applications. This review aims to summarize the recent advancements in nanoparticle-based drug delivery to control ER stress where the ER and mitochondria are involved, providing valuable insights for the design and efficient delivery of drugs targeting these subcellular organelles.

## 2. Role of endoplasmic reticulum and mitochondria

**Figure 1.**
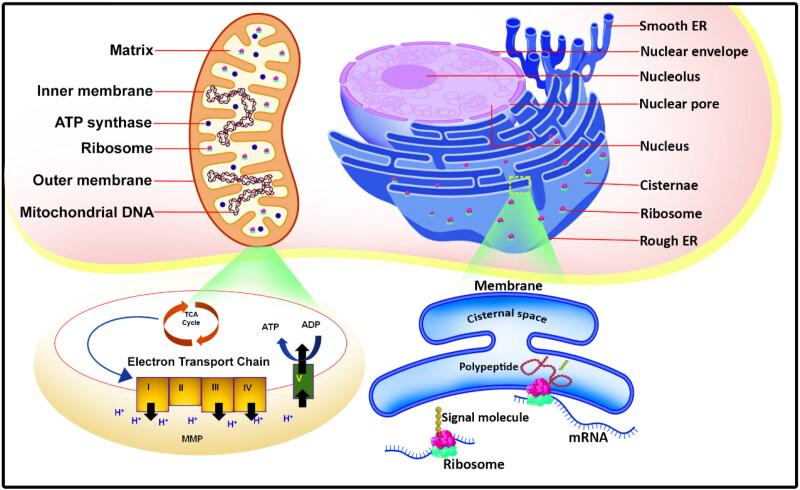
Schematic representation of structure and function of endoplasmic reticulum and mitochondria.

**Figure 2.**
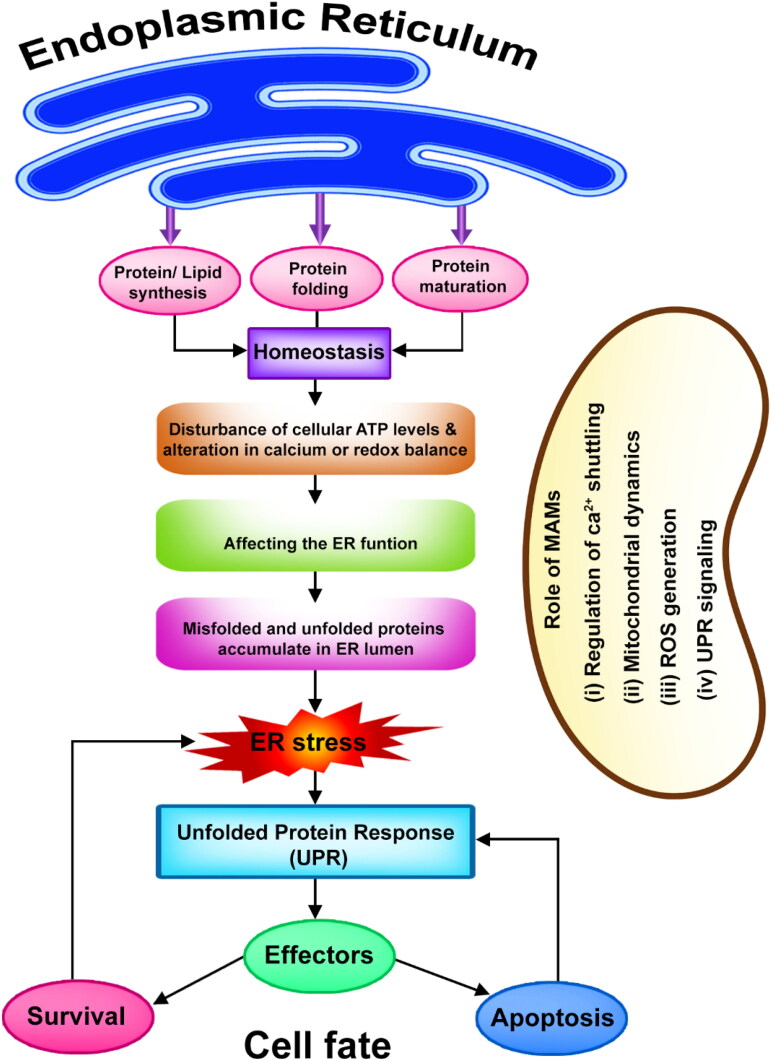
The major roles of endoplasmic reticulum and route to ER stress dependant unfolded protein response.

ER is a highly intricate reticular system present in eukaryotic cells exhibiting diverse morphologies (Lam & Galione, 2013). It occupies a significant fraction of the cell’s membrane area and comprises distinct regions, including the nuclear envelope, rough ER, and smooth ER (Figure 1). The ER plays essential roles in various cellular processes, such as lipid and sterol synthesis, intracellular calcium regulation, and the modification and quality control of proteins after translation (Shen et al., 2004; Luarte et al., 2018). In the ER, proteins are biosynthesized, folded, modified, and assembled, among other functions. It houses ribosomes that synthesize a significant portion of cellular proteins, which then acquire proper folding in the ER lumen before being transported to their destinations. The ER also performs post-translational modifications on proteins and serves as a calcium reservoir, dynamically regulating intracellular calcium levels. These functions of the ER contribute to cellular homeostasis and functionality. Disruptions in ER homeostasis can arise from genetic and environmental factors, leading to stress referred to as endoplasmic stress (Figure 2). Proteins that are unfolded or misfolded accumulate within the ER lumen, resulting in this stress. In reaction, cells trigger the unfolded protein response, a self-regulating mechanism initiated by three transmembrane proteins positioned in the ER: inositol-requiring enzyme 1α, PRKR-like (interferon-induced dsRNA-activated protein kinase) ER kinase, and activating transcription factor 6 (Table 1). These proteins detect ER stress and coordinate a response to alleviate moderate stress levels. While the unfolded protein response can help alleviate moderate ER stress, prolonged or severe stress can trigger programmed cell death. This process is implicated in the development of numerous diseases, including cancer, neurodegenerative disorders, metabolic disorders, and cardiovascular diseases. Understanding the intricate relationship between ER function, ER stress, and the unfolded protein response is crucial for comprehending the pathogenesis of these diseases. Targeting the unfolded protein response pathways and restoring ER homeostasis holds the potential for therapeutic interventions in the treatment and management of these conditions.

**Table 1.** Important protein-complexes and pathways involved in ER stress.

Players and pathways in ER stress	Functions	References
Rough endoplasmic reticulum (RER)	Biosynthesis of proteins in the presence of membrane-bound ribosomes that includes post-translational modifications, protein folding, and sorting.	(Voeltz et al., 2002)
Smooth endoplasmic reticulum (SER)	Synthesis and storage of lipids and steroids	(Bardo et al., 2006)
Unfolded protein response (UPR)	ER stress triggered the reestablishment of protein homeostasis.	(Read & Schröder, 2021; Deka et al., 2022)
Inositol requiring enzyme-1 (IRE1)	Type-1 transmembrane protein of ER, UPR signaling transducer, ER stress sensing by N-terminal luminal domain	(Zhu et al., 2014)
Protein kinase RNA-like ER kinase (PERK)	ER resident protein, main transducers of ER stress, misfolded protein synthesis inhibited by activated PERK due to phosphorylation of eIF2α, PERK-eIF2α pathway promotes autophagy & apoptosis.	(Moon et al., 2016)
Activating transcription factor 6 (ATF6)	Type-II transmembrane protein of ER, During ER stress, ATF6 translocates to Golgi after detachment of GRP78 for site-1 & 2 proteases driven cleavage. The cleaved ATF6P50 moves to the nucleus to regulate the expression of CHOP & XBP1 (X-Box Binding Protein-1).	(Simard et al., 2015)
Glucose-regulated protein-78 (GRP78) or Binding immunoglobin protein (BiP)	Molecular chaperone, belonging to the HSP70 family, compels the unfolded or misfolded protein to refold or degrade.	(Girona et al., 2019)
C/EBP homologous protein (CHOP)	Regulates the expression of proapoptotic and anti-apoptotic genes like Bcl2, UPR downstream effector induce apoptosis.	(Shimodaira et al., 2014)

An inner and outer mitochondrial membrane, an inner mitochondrial membrane space, along with the mitochondrial matrix are the major components of mitochondria (Lan et al., 2019). In addition to providing energy to cells, mitochondria allow the tricarboxylic acid cycle and oxidative phosphorylation to take place in it and participate in apoptosis, cell differentiation, and signaling (Figure 1) (Ghosh & Girigoswami, 2008). The mitochondrial three levels of the quality control mechanism are an internal security mechanism (Franco-Iborra et al., 2018). During mild stress conditions in the cell, they are capable of maintaining protein homeostasis along with structural integrity at molecular and organellar levels to establish correct functionalities. However, when the stress is too severe, mitochondria begin to apoptose to make sure that mitochondrial quality control is maintained. The physiological and pathological functions of the ER and mitochondria are highly interconnected. In response to stress, mitochondria and ER functions can be coordinated to reinstate cellular homeostasis (Bilen et al., 2022; Giorgi et al., 2009). The presence of mitochondria-associated membranes (MAMs) in close proximity to the ER membranes has long been recognized as a way in which ER membranes can form lipid raft-like domains that are similar to mitochondrial membranes (Raturi & Simmen, 2013). ER, and mitochondria of a cell are physically connected by MAMs, which play a crucial role in the process of lipid biosynthesis and the transfer of calcium ions from the ER to the mitochondria via MAMs.

## 3. Unfolded protein response and mitochondria

UPR regulation and mitochondrial function are linked by several regulatory mechanisms. Mitochondrial regulators such as ATF-4 are affected by UPR transducers and Parkin, an important regulator of mitochondrial activity and dynamics (Senft & Ronai, 2015). The sXBP1 (spliced XBP-1) pathway is activated by Parkin and exhibits reciprocal activation through UPR signaling. In essence, Parkin represents a regulatory node of the UPR which is involved in crosstalk with ATF-4 when activated. Additionally, Parkin appears to modulate mitochondrial bioenergetics by increasing MAMs during ER stress by maintaining calcium transfer (Ca^2+^) between ER and mitochondria (Gong et al., 2021). It has been noticed that Parkin is responsible for the mitophagy of impaired or malady mitochondria, but this has not been proven. The Parkin-driven mitophagy occurs when unfolded or misfolded proteins are generated to activate mitochondrial UPR and increase the expression of the mitochondrial chaperone (Iorio et al., 2021). ER stress, UPR, and mitochondrial dysfunction are linked by additional mechanisms, including ER and UPR cues that regulate mitochondrial fusion and fission processes. Activating mitofusin-2, genetically promotes mitochondrial swelling, excess Ca^2+^, and reduced respiration by preventing the fusion process from occurring (Chen & Chan, 2009; Rovira-Llopis et al., 2017).

Munoz et al. have shown that both mitofusin-2 and PERK are important regulators of mitochondrial functions (Muñoz et al., 2013). It was therefore found that PERK inhibition can rescue mitochondrial integrity in mitofusin-2 mutant cells. Correspondingly, both mitofusin-2 and PERK were found to control the MAM principle or integrity. In spite of the fact that PERK may be able to regulate mitochondrial dynamics independently of its UPR function, UPR-reliant mechanisms cannot be excluded (Verfaillie et al., 2012). It may be implicated that MAM integrity may play a role in UPR signaling due to its hyperactive UPR signaling process and, flawed autophagy, and hampered apoptosis in mitofusin-2 deficient cells. In a tissue-specific manner, the transcriptional coregulatory PGC1α (peroxisome proliferator-activated receptor gamma coactivator-1 alpha) is connected to the UPR in order to influence mitochondrial biogenesis and function. ATF-6, in conjunction with PGC1α mediates an adaptive response to stress in skeletal muscle on ER stress during rigorous exercise. Therefore, the UPR has several avenues of influence on mitochondrial bioenergetics. One is that it promotes mitophagy, which helps to remove stressed mitochondria, and the second is that it influences the MAM, which regulates mitochondrial bioenergetics. ER stress pathways and calcium level dysregulation create a pathological assessment or feedback loop between mitochondria and ER that promotes disruption of both organelles and causes several complications or diseases (Andhavarapu et al., 2020).

## 4. Disease-associated with ER and mitochondria dysfunction

### 4.1. Cancer

The development of cancer primarily occurs due to genetic mutations in crucial regulatory genes, resulting in cell activation and the acquisition of a more resilient and aggressive phenotype (Qin et al., 2020). Tumor cells exhibit abnormal proliferation and metabolism, disrupting the balance of their surrounding environment and giving rise to the tumor microenvironment. The microenvironment is characterized by conditions such as nutrient deficiencies, accumulation of reactive oxygen species, acidosis, and hypoxia (Mortezaee & Majidpoor, 2023). Although these conditions are detrimental to normal cells, they create a favorable environment for tumor cells to thrive, evade the immune system, and metastasize. Additionally, the tumor microenvironment can induce alterations in infiltrating immune cells, further worsening the immunosuppressive environment. This aggravation of immunosuppression promotes tumor progression and impairs the immune system’s ability to effectively detect and eliminate cancerous cells (Hosseinikhah et al., 2023). To develop effective therapeutic strategies targeting tumor growth and metastasis, it is crucial to have a comprehensive understanding of the complexities of the tumor microenvironment. By manipulating this microenvironment and overcoming immunosuppression, it may be possible to enhance the efficacy of cancer treatments and improve patient outcomes (Singh et al., 2016).

**Figure 3.**
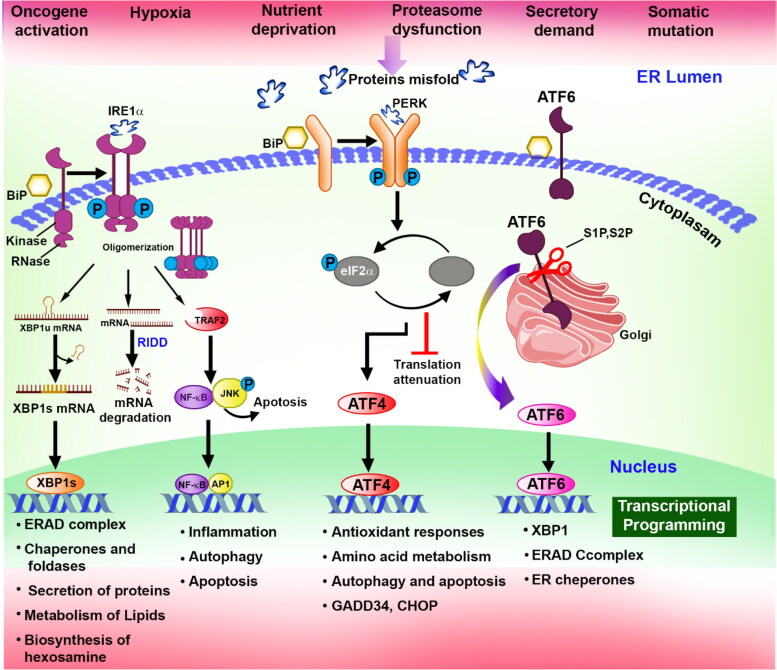
The diagram focuses on how UPR functions in cancer and how they are affected by extrinsic stress that can lead to protein folding in the endoplasmic reticulum.

Tumor cells face numerous obstacles arising from both external and internal factors. External challenges include the hostile tumor microenvironment, while internal challenges arise from the high demand for protein synthesis and folding within the ER (Chen et al., 2020). To survive and adapt, tumor cells activate distinct signaling pathways that are associated with the unfolded protein response (Figure 3). These pathways enable tumor cells to efficiently detect and respond to ER stress, facilitating the restoration of cellular equilibrium (Rashid et al., 2015). In this connection, the role of Myc oncogenes is important that it initiates and helps in the progression of malignancies through the modifications of unfolded protein responses. Moreover, Myc enhances a load of proteins in the ER by elevating the expression of mRNA, and at the same time, it binds to several activated transcription factors to make complexes that affect the ER stress (Parker et al., 2001). Under harsh microenvironments, tumor cells are fine-tuned to thrive under ER stress, and targeting aggravated ER stress or perturbed unfolded protein response can be a favorable strategic therapy against cancer progression through pharmacological intervention or gene regulations.

**Figure 4.**
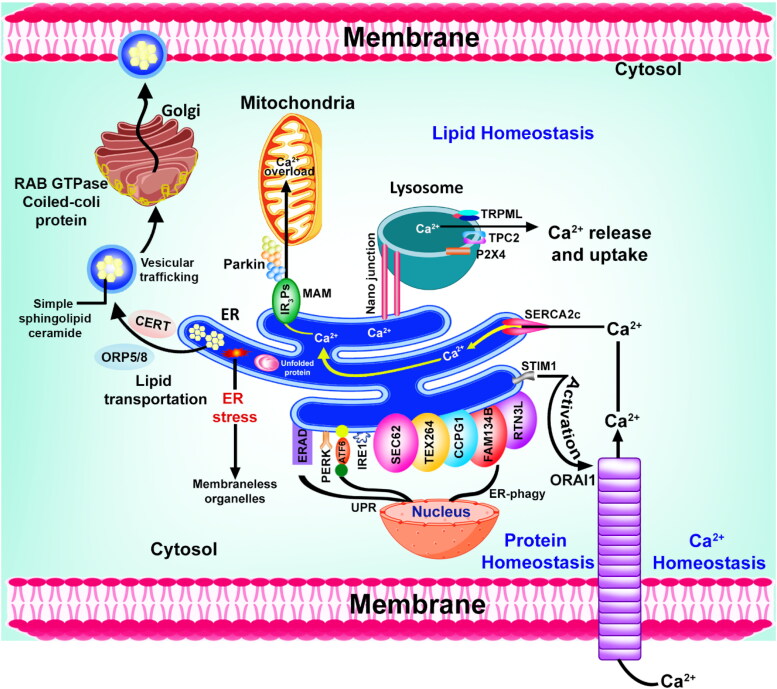
Schematically represented how the lipids, proteins, and calcium are metabolized and homeostasized by the ER due to direct or indirect communication with other cell organelles.

Research findings indicate that a significant portion of the mitochondrial surface, nearly 5-20%, comes into direct contact with the ER (Rizzuto et al., 2009). The MAM plays a crucial role in promoting biogenesis by synthesizing phospholipids as well as sphingolipids (Figure 4). Notably, in cases of mitochondrial dysfunction, many cancer cells exhibit distinct alterations in lipid metabolism, such as lipid-free biosynthesis and a lipogenic phenotype. This suggests a close relationship between mitochondrial dysfunction and lipid-related changes in cancer cells. Consequently, studies have indicated that tumorigenesis, migration, invasion, and cell survival are influenced by mitochondrial and lysosomal dysfunction, as well as ER stress-related pathways (Lin et al., 2019). Furthermore, mitochondrial dysfunction has been identified as a platform for various cellular signaling pathways, including oncogenic signaling. Additionally, lysosomal dysfunction has been found to enhance apoptosis induced by oxidative stress, primarily through the accumulation of ubiquitinated proteins in cancer cells (Li et al., 2016). There have been numerous reports suggesting that the UPR is activated in a variety of cancers in humans as well as in multiple numbers of animal and cellular models of cancer. It is often observed that UPR markers are upregulated in tumors, which indicates the presence of ER stress (Fan et al., 2018). Several studies have shown that the UPR may also contribute to cancer independently of protein misfolding in hypoxic tumouric conditions; however, several studies demonstrated that the UPR plays a role in its development as well. GRP78 and GRP94, which are two classic UPR markers, are often highly expressed in solid tumors, indicating an elevated level of ER chaperones (Hiss & Gabriels, 2009; Siegelin, 2012). Various ER stress markers and their study models are shown in Table 2.

**Table 2.** ER stress markers used to study different types of cancers.

Type of cancer	Cells/ tissues	ER stress marker	Therapeutics used	Ref.
Ductal breast carcinoma or ductal carcinoma in situ (DCIS)	Tissue collected from 250 no. of DCIS patient	Elevated level of GRP78 & CHOP	Anthracycline	(Zheng et al., 2014)
Breast cancer	BT474, MCF-7, MCF-10A, MDA-MB-23, THF-1 breast cancer cell lines	miR-27a-3p, PD-L1	–	(Yao et al., 2020)
Breast cancer	MCF-7, MDA-MB-231/453/468, SKBR-3, T47D	RNF5 controlling L-glutamine transporters SLC1A5/38A2	Paclitaxel	(Jeon et al., 2015)
Breast cancer	MCF-7/ADM cell line	TM9SF4	Adriamycin	(Zhu et al., 2019)
Breast cancer	MCF-7, MDA-MB-231	BiP, IRE1α, GADD153, Ca2+ level in cytoplasm, Bcl2 level.	Usnic acid derivative isoxazole	(Pyrczak-Felczykowska et al., 2022)
Triple negative breast cancer and prostate cancer	HCC2185, MDA-MB-453, MFM-223 cell for LAR TNBC and LNCap & 22RV1 cell line for prostate cancer	Androgen receptor expression, PERK/eIF2α/ATF4 signaling	Thapsigargin & brefeldin A	(Li et al., 2022)
Prostate cancer	LNCaP & CWR22Rv1	EDEM3 expression	Thapsigargin & tunicamycin	(Scott et al., 2022)
Castration-resistant prostate cancer	Prostate cancer cells	c-Jun N-terminal kinase (JNK)	Flavonoids compound gambogenic acid, autophagy inhibitor chloroquine, 4-phenylbutyric acid as JNK blocker, ROS scavenger N-acetyl-L-cysteine	(Wu et al., 2023)
Prostate cancer	PC3 & DU145 cell lines	MMP, expression level of IRE1α, ATF-6α and phosphor-eIF2α	Hesperidin flavonoid	(Jeong et al., 2022)
Oral squamous cell carcinoma	OSCC tumor tissues, HN4 cell lines	PD-L1 expression, PERK, ATF-6, GRP78	Interferon gamma	(Yuan et al., 2022)
Ovarian cancer	Epithelial OVcar3, SKov3 ovarian cancer cells	Calreticulin	Doxorubicin, ER stressor thapsigargin and inhibitor TUDCA	(Abdullah et al., 2022)
Colorectal cancer (CRC)	SNU-C5/5-FUR	GRP78, PERK, eIF2α, IRE1, XBP-1, Caspase-12, CHOP	5-fluorouracil, shikonin, ER stress inhibitor TUDCA	(Piao et al., 2022)
Lung cancer	A549 & NCI-H292	MMP, dysregulation of Bax/Bcl-2, Cytoplasmic vacuolation, expression of Alix, GRP78, IRE1, PERK, ATF-6	Kuwanon	(Ma et al., 2023)
Liver cancer	Hepatocellular carcinoma cells, mTHP-1 & RAW264.7 cells	CD68^+^, miR-23a-3p exosomes driven expression of PD-L1, GRP78, ATF-6, PERK	–	(Liu et al., 2019)
CRC	WS480, HCT-116, HT-29 and normal NCM-460 colonic epithelial cells	BiP, PERK, eIF2α, CHOP, ATF-4	Withaferin-A & 5-fluorouracil	(Alnuqaydan et al., 2020)
Castration-resistant prostate cancer	DU145 & PC3 and normal prostate epithelial RWPE-1 cell lines	BiP, eIF2α, PDI, p-eIF2α, IRE1α	δ-TT (tocotrienols)	(Fontana et al., 2019)
Breast cancer	293T, MCF-7, MDA-MB-468	GRP78, lncRNA-MIAT, OCT4, AKT	5-fluorouracil	(Yao et al., 2020)

**Table 3.** Engineered nanomaterials targeting ER for potential applications.

	Engineered nanoparticles	Mode of actions	Applications	Ref.
1.	Self-assembled ER-targeting graphene oxide nanoparticles	Promotes ER stress-related apoptosis in lung cancer, breast cancer, and multidrug-resistant triple-negative breast cancer (TNBC)	Promising therapeutic tool in cancer by exploiting ER stress and UPR	(Pandey et al., 2020)
2.	Liposomal nanoformulation of calcium channel blocker azelnidipine along with medroxyprogesterone acetate	Induces acute ER stress and proapoptotic genes upregulation to interfere DNA replications for promoting cell death	The nanoformulation ruined calcium homeostasis to activate acute ER stress for the treatment of endometrial cancer	(Huang et al., 2022)
3.	Thapsigargin encapsulated in PLGA nanoparticles	Nanoformulation induces autophagy and UPR pathway in human kidney tubular epithelial cells (HK-2) and protects them from oxidative stress.	Favorable approach for the prevention of chronic kidney disease	(Cheng et al., 2019)
4.	Protein disulfide isomerase CCF642 entrapped in albumin nanoparticles in combination with temozolomide	Nanoparticle-based therapeutics induce ER perturbation by down-regulating PERK signaling, which triggers cell death beyond repair.	Remarkable reduction of orthotopic tumor growth	(Kiang et al., 2023)
5.	Silica nanoparticles	Apoptosis, oxidative and ER stress were all associated with silica nanoparticles-induced vascular injury in arteries.	Atherosclerosis is potentially preceded by endothelial dysfunction caused by silica nanoparticles	(Li et al., 2019)
6.	ER targeted PdPtCu nanozyme	Reprogram tumor microenvironment (TME), activates the antitumor immune response and IDO-driven immune escape by NLG919.	The killing of tumor cells by PDT, PTT, and chemodynamic therapy (CDT)	(Xie et al., 2023)
7.	Zn-ferrite nanoparticles to target FAP^+^ (fibroblast activating protein positive)	ER stress and mitochondrial damage intensified by magnetocaloric effect under alternating magnetic field.	Potential tools to treat rheumatoid arthritis	(Qi et al., 2023)
8.	Dual targeting nanoparticles made of P(ERMA-*co*-DMA)-b-PCSMA and PDMA-*b*-PCSMA	Upregulate IRE1α & CHOP, boosting Ca^2+^ efflux and activating caspase-12 cascade.	Useful for cancer theranostic in precision healthcare	(Wang et al., 2023)
9.	Methotrexate and diacerein-loaded solid lipid nanoparticles	Alter ER stress-mediated apoptosis	Promising therapeutics for rheumatoid arthritis	(El-Refaie et al., 2023)
10.	CdTe quantum dots	ROS generation and prolonged ER stress to activate PERK and autophagy	Apoptotic death of hepatocellular carcinoma	(Zhang et al., 2023)
11.	Redox-responsive phosphorus dendrimer-Cu complex and toyocamycin-entrapped polymeric nanoparticles coated with membranes of cancer cells	Apoptosis and immunogenic cell death	Synergistic chemotherapy-enhanced immunotherapy effects against various types of tumors	(Guo et al., 2022)
12.	Size dependant iron oxide nanoparticles	Elevate the neutrophils and IL-6 to induce tumor necrosis factor-α	Help to study the biosafety of iron oxide nanoparticles to protect human health	(Ying et al., 2022)
13.	Hydroxylated [70] fullerene nanoparticles	Repress the JNK to reactivate the insulin receptor substrate signaling pathway and inhibit gluconeogenesis.	Applicable to diseases related to insulin resistance	(Li et al., 2022)
14.	Theranostic nanocomposites made of AgNPs and peptide-functionalized DOX	Induce organelle-driven immonochemotherapy and drug efflux protein diffidence	Theranostic agents against drug-resistant breast cancer	(Jiang et al., 2022)
15.	Se nanoparticles synthesized using *Lactobacillus casei*	Alleviate oxidative stress, damage to ER structure, and activation of PERK.	A potential solution to prevent mycotoxins like deoxynivalenol	(Song et al., 2023)

While conventional treatments like cytotoxic drugs and radiation can trigger some degree of ER stress, this modest disruption often proves ineffective against specific types of tumors, such as renal cell carcinoma. In some cases, these therapies can even promote oncogenic activity due to the activation of signaling pathways associated with the adaptive UPR (Cai et al., 2021). Additionally, these nonspecific interventions can lead to severe systemic toxicity. As a result, these shortcomings hinder the widespread adoption of clinical therapies focused on inducing ER stress. Plenty of nanoparticles like Ag, ZnO, and Fe_3_O_4_, which are intrinsically capable of inducing ER stress, may offer promising alternatives to promote ER stress at the site of the tumor (Jang et al., 2013; Amsaveni et al., 2013; Girigoswami et al., 2018). Furthermore, these nanoparticles not only play the role of targeted nanocarriers, but they also serve to rattle the balance of an ER lumen’s redox state, thereby disrupting the folding of proteins and elevating the level of ER stress. In particular, Fe_3_O_4_ nanocrystals, which are dual enzyme mimetic nanoparticles, are able to redox target tumors based on the metabolic microenvironment that differs between tumors and healthy tissues (Yang & Shi, 2020). In neutral environments, such as blood circulation and normal tissues, Fe_3_O_4_ nanocrystals function as an enzyme catalase to break down H_2_O_2_ into water and oxygen, whereas in the acidic microenvironment, it provokes Fenton reaction as peroxidase. In addition, Fe_3_O_4_ nanoparticles are capable of laser-induced photothermal generation of ROS that causes ER oxidative damage, and their magnetic properties help in magnetic hyperthermia to raise ER stress in an acid tumor microenvironment (Neha Desai et al., 2021). Therefore, nanoparticles and nano-enabled drug delivery systems can improve the efficacy of antitumor drugs by targeting ER stress-related regulatory factors and effectors.

### 4.2. Pathogenesis of neurodegenerative disorder

**Figure 5.**
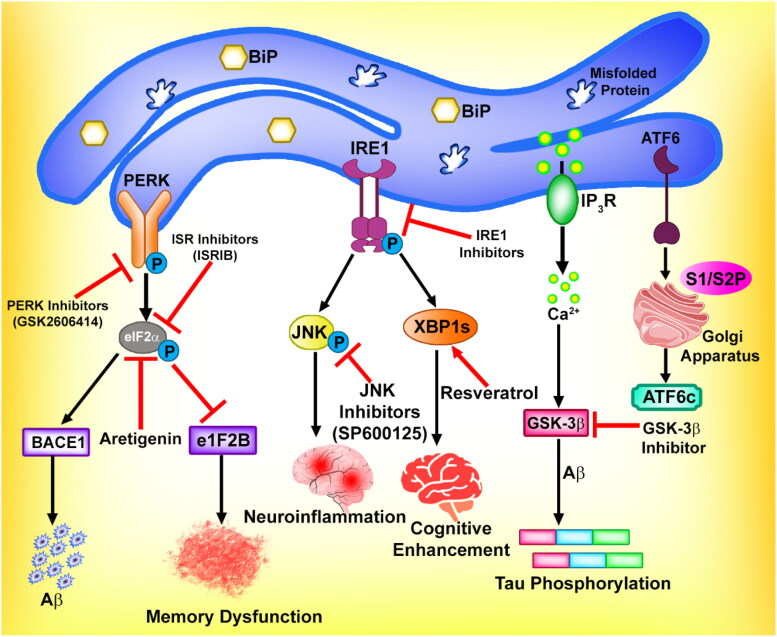
Targeting ER stress and UPR to control neurodegenerative disorder.

Several research findings suggest a strong connection between neurodegenerative disorders or anarchy, such as Alzheimer’s disease, Creutzfeldt–Jakob disease (CJD) or prion disease, and Parkinson’s disease, and the generation of excessive ER stress in neuronal cells (Harini et al., 2022; Agraharam et al., 2022). Disruption in the ER functions causes an accumulation of unfolded or misfolded proteins in neurons, triggering ER stress and potentially resulting in neurotoxicity. When ER stress is moderate, the unfolded protein response can effectively mitigate the stress. However, higher ER stress disturbs the cellular balance leading to progressive deterioration of neuronal function, whereas chronic ER stress or the terminal state triggers programmed cell death (Figure 5).

#### 4.2.1. Alzheimer’s disease (AD)

AD is a prevalent neurodegenerative disorder that becomes more common with age and contributes significantly to reported cases of dementia. The underlying pathophysiology of AD involves the development of neurofibrillary tangles intracellularly, composed of hyperphosphorylation of tau, as well as the accumulation of amyloid-β aggregates extracellularly (Harini et al., 2022; Agraharam et al., 2022). Tau is a protein normally responsible for stabilizing microtubules and facilitating cellular processes like vesicle trafficking and elongation of axons along with mutation. Studies have shown that reduced tau levels lead to significant impairments in neurite growth in cerebellar neurons. However, certain factors disrupt the balance between tau kinases and phosphatases, leading to abnormal tau phosphorylation. In its hyperphosphorylated form, tau becomes soluble and polymerizes into oligomers and neurofibrillary tangles. The soluble form of tau protein can initiate pathological ER stress by disrupting key components involved in ER-associated protein degradation (ERAD) (Cai et al., 2016). Interestingly, it has been observed that preexisting ER stress can also promote the formation of neurofibrillary tangles. It is widely recognized that ER stress responses triggered by amyloid-β can activate various kinases, including glycogen synthase kinase 3, which can subsequently phosphorylate specific sites on tau (Lambourne et al., 2005; Mandlik et al., 2022). The phosphorylation process plays a role in the development of neurofibrillary tangles. As a result, there exists a reciprocal relationship between ER stress and hyperphosphorylated tau, where they can induce each other in a cyclic manner, thereby propagating the progression of AD. Nevertheless, recent scrutiny has shed light on the correlation between neurofibrillary tangles formation and the severity of AD. Current evidence suggests that soluble oligomers of tau and amyloid-β may serve as the primary neurotoxic agent that contributes to AD (Singh et al., 2023).

#### 4.2.2. Parkinson’s disease (PD)

PD is an ongoing and advancing neurological condition marked by the gradual decline of specific dopamine-producing neurons within the substantia nigra, which is a region situated in the brain (Sanyal et al., 2016; Ahmed et al., 2016). A distinguishing feature of PD is the presence of Lewy bodies, which are abnormal protein clumps found inside nerve cells. Although the exact cause of Lewy body formation is not fully understood, they are widely recognized as key pathological features of PD. Research conducted after death has revealed that a higher percentage of dopaminergic neurons containing Lewy bodies also show signs of caspase-3 activity, an enzyme associated with cell death, compared to dopaminergic neurons without Lewy bodies (Özduran et al., 2022). This suggests that the presence of Lewy bodies increases the vulnerability of dopaminergic neurons to undergo apoptosis, a process of programmed cell death. The principal component of Lewy bodies is an abnormal protein called α-synuclein, which is believed to form due to various genetic factors, including aberrant post-translational modifications (O'Hara et al., 2020). Mutations commonly associated with autosomal recessive PD are often found in the Parkin gene. This particular gene is responsible for producing an E3 ubiquitin ligase that plays a crucial role in the process of mitophagy. Findings from studies utilizing brain samples have provided evidence that Parkin, an important gene implicated in autosomal recessive PD, can be inactivated through various post-translational modifications, including the addition of dopamine (Vrettou & Wirth, 2022). A more accepted theory is that the E3 ligase activity of Parkin or encountering defects in PINK1 gene, which is responsible for recruiting Parkin to the outer membrane of damaged mitochondria, plays a significant role in the development of PD and the inability to maintain mitochondrial integrity (Han et al., 2023; Zhang et al., 2022). Additionally, it has been observed that mutation in LRRK2 (leucine-rich repeat kinase-2) gene also contributes to the promotion of PD. Findings obtained from several studies indicate the presence of abnormal α-synuclein could potentially worsen the advancement of this disease by triggering excessive or uncontrolled ER stress counters. The unfolded protein response induces the expression of Parkin as a defensive mechanism against stress. By preventing the toxic buildup of Parkin substrates, the augmented Parkin protein level ensuring from this response safeguards neurons against cell death induced by ER stress (El Manaa et al., 2021). Additionally, the protective role of Parkin can be attributed, in part, to its ability to enhance the generation of sXBP-1, a transcription factor with a basic region of leucine zipper structure that activates the expression of genes associated with cell survival. Recent research findings further suggest that Parkin regulates the functioning of PS1 and PS2, raising the possibility of the potential connection between faulty Parkin and the progression of PD as well as AD (Himalian et al., 2022; Chatanaka et al., 2023).

#### 4.2.3. Prion disease

Prion disease, which is alternatively cited as transmissible spongiform encephalopathies (TSEs), encompasses a group of transmissible neurodegenerative disorders (Legname & Scialò, 2020; De Pedro-Cuesta et al., 2021). Examples of these conditions include CJD, BSE (bovine spongiform encephalopathy or mad cow disease), and scrapie. TSEs exhibit distinct pathological characteristics, primarily characterized by the building up of misfolded and resistant variants of the PrP (prion protein) within the brain (Thüne et al., 2019). As the TSEs disease advances, it leads to the degeneration of brain tissue in a spongiform pattern. This degeneration is accompanied by heightened astrogliosis and extensive neuronal apoptosis. PrP^Sc^, the pathological variant of PrP, remains unchanged in terms of its amino acid sequences and post-translational modifications when compared to the normal PrP. However, an important distinction arises as PrP^Sc^ undergoes a conformational alteration form α-helical forms, resulting in an elevated presence of β-sheeted secondary structures (Prusiner, 1998). There has been a proposition that the escalation of PrP^Sc^ could potentially stabilize misfolded subtypes within the ER, subsequently causing toxicity through various sig­naling pathways like caspase activation, Ca-dysregulation, autophagy, and ER stress. This ER stress performs a notable role in the evolution of prion disease by promoting the accumulation of PrP^Sc^ as well as enhancing the susceptibility of the wrongly folded form of PrP^C^ to undergo prion alteration.

#### 4.2.4. ER stress in diabetes mellitus (DM)

Type-1 and type-2 are the two primary variations of DM, having a significant impact on the well-being and longevity of numerous individuals, adversely affecting both their quality of life and life span (Sathyaraj et al., 2023). One crucial aspect of type-1 DM involves a decrease in the mass of β-cells primarily attributed to heighten β-cells apoptosis and defective β-cells regeneration. Type-1 DM is distinguished by a significant deficiency in insulin generation, primarily sourced by the targeted annihilation of pancreatic β-cells, a process that generally occurs gradually over a span of multiple years (Tan et al., 2019). The loss of β-cells in type-1 DM is attributed to an autoimmune-driven mechanism, in which chronic inflammation referred to as insulitis leads to the destruction of β-cells. This destructive process is facilitated by the release of cytokines and other substances by invading immune cells, as well as the expression of certain factors on their cell surfaces. These immune-mediated actions initiate secondary pathways that induce cell death, specifically in the targeted β-cells (Cardinal et al., 2001). In the context of type-2 DM, this decline in β-cells mass is frequently backed by insulin resistance in adipose tissue, muscle, and the liver. Type-2 DM arises from a decline in the pancreatic β-cells’ ability to secrete an adequate amount of insulin necessary for promoting glucose uptake in peripheral tissues. As the capacity of secretion of β-cells diminishes, glucose tolerance deteriorates, resulting in progressively elevated fasting glucose levels that eventually lead to evident hyperglycemia. The development of type-2 DM involves impairments in both insulin secretion and mechanism of action, although it is now recognized that insulin deficiency plays a crucial role, as the absence of this deficiency prevents the onset of type-2 DM (Mariadoss et al., 2022). This deficiency in β-cell affair is present early in the progression of the disease and can be detected by significantly downsized first-phase or intense glucose-induced insulin secretion. Emerging research suggests that the depletion of β-cells in both type-1 DM and type-2 DM is triggered by stress responses coordinated by essential transcription factors and networks of genes (Montane et al., 2014). These initial signaling pathways often converge into shared ‘execution’ pathways, including ER stress, mitochondrial dysfunction, and the generation of ROS. Additionally, ER stress may serve as a connection between obesity, and the resistance of insulin in the liver and adipose tissue, thereby suggesting that this cellular response could potentially serve as a unified mechanism underlying both β-cell dysfunction and impaired insulin signaling in type-2 DM (Amen et al., 2019; Mandal et al., 2021).

Many of the authorized medications designed to address cognitive impairments related with neurodegenerative disorders rely on the modulation of neurotransmitters or enzymes, such as acetylcholinesterase inhibitors (Cheng et al., 2023; Wechsler et al., 2019). Nevertheless, these therapeutic approaches often encounter setbacks due to issues like poor drug solubility, limited bioavailability, and difficulties traversing the blood-brain barrier. To enhance drug transport to the brain, researchers have devised numerous nano-enabled platforms aimed at overcoming the challenges posed by the blood-brain barrier and the opsonization process triggered by plasma proteins within the systemic circulation. These nanoscale systems facilitate the passage of drugs through the blood-brain barrier, ultimately enhancing drug bioavailability, pharmacokinetics, and pharmacodynamics. Notable examples of such drug carriers include dendrimers, hydrogels, polymeric nanoparticles, solid-lipid nanoparticles, and lipid-based nanoparticles. Prior research has demonstrated that nanoparticles with a diameter of ≤ 200 nm exhibit more efficient internalization, deeper brain penetration, and prolonged circulation compared to their larger counterparts (Tsou et al., 2017). However, nanoparticles with a diameter of ≤ 5 nm are susceptible to rapid renal clearance, coupled with limited drug loading capabilities and swift release kinetics, rendering them unsuitable as drug delivery vehicles. Within this size range, nanoparticles demonstrate a superior ability to traverse biological barriers, maintain circulation, and target the desired cells effectively. Depending on the specific properties of the materials involved, nanoplatforms have the capability to react to natural bodily triggers, such as reactive oxygen species, changes in pH levels, and oxygen deficiency. A range of responsive nanoplatforms utilizing various materials, including structures reminiscent of LEGO blocks that rely on cyclodextrin, cucurbiturils, and other supramolecular components (Yue et al., 2020; Zhou et al., 2021). These structures release their enclosed medications when they encounter specific disease-related proteins. Additionally, certain metal–organic frameworks can release drugs when exposed to acidic conditions due to the disruption of covalent bonds (Yue et al., 2020). In the context of AD, pathological alterations like the excessive buildup of metal ions, ROS, and amyloid-beta offer ideal environmental cues for these responsive nanoplatforms. Furthermore, these responsive systems hold the promise of intelligent drug delivery, adapting to dynamic changes in the pathological microenvironment and avoiding interruption to microenvironmental homeostasis caused by excessive treatment.

#### 4.2.5. ER stress in liver disease

In addition to performing metabolic functions, the liver also secretes and excretes substances. Hepatocytes, the primary cell type in the liver, have significant responsibility for synthesizing and secreting various proteins such as albumin, alpha-1 antitrypsin, and lipoproteins (Nguyen et al., 2020). Given the hepatocytes’ high demand for protein synthesis and folding, they possess an abundance of ER and are susceptible to disruptions and stress in the ER. A protective cellular mechanism known as the unfolded protein response is activated to restore the homeostasis of ER and promote cell survival in response to ER stress. In addition to its contributing role in the synthesis of protein, the ER is a key site for lipid metabolism in hepatocytes. Triglycerides, the primary components of fats, are primarily synthesized from fatty acids and glycerol within the ER. Furthermore, the ER serves as the assembly site for very low-density lipoprotein before it is transported to the Golgi apparatus. Thus, maintaining ER homeostasis is closely linked to hepatic lipid balance. Research has shown that ER stress can induce hepatic steatosis or the accumulation of fat in the liver (Jia et al., 2022; Luo et al., 2022). However, if the restoration process fails, prolonged ER stress and unfolded protein response activation can lead to cell death. Thus, various liver diseases in humans may be caused by ER stress and unfolded protein responses. Fat deposits exceeding 5% of liver mass define nonalcoholic fatty liver disease as a clinical pathophysiological syndrome. Hepatocytes that possess a plentiful smooth endoplasmic reticulum have a crucial function in the production and movement of fatty acids and cholesterol. It is believed that an ER stress response occurs due to factors such as a fatty diet, obesity as well as insulin resistance. This stress response disrupts the process of lipogenesis and exacerbates the accumulation of fat in the liver, resulting in a vicious cycle that drives the advancement of fatty liver disease toward more severe conditions like hepatitis, hepatic fibrosis, and even hepatic cirrhosis. The unfolded protein response is activated as a response to ER stress, serving as a protective mechanism for the liver during the early phase (Das et al., 2023). However, as the stress persists, UPR can intensify inflammatory responses, leading to the promotion of hepatocyte apoptosis. Hepatocytes are the primary location where the ER-localized Cytochrome P450 (CYP2E1) is predominantly found (Shi et al., 2022). This particular enzyme, CYP2E1, is a monooxygenase that displays high inducibility when exposed to ethanol. Persistent alcohol consumption is linked to an increased expression of hepatocyte CYP2E1, which enhances the oxidative breakdown of ethanol and results in the excessive accumulation of toxic metabolic byproducts, particularly acetaldehyde along with ROS. This process contributes to ER stress and the development of alcoholic liver damage (Song et al., 2020; Liu et al., 2021). However, it is noteworthy that the initiation and perpetuation of alcoholic liver damage primarily involve oxidative stress mediated by mitochondria rather than ER stress. It is important to acknowledge that the ER and mitochondria have a close association both in terms of their physiological architecture and biological and biochemical functions.

In recent studies, scientists have explored strategies to mitigate liver damage by targeting the ER stress pathway. For instance, they investigated the effectiveness of 4-PBA (4-phenyl butyric acid), an inhibitor of ER stress, in alleviating cholestatic liver disease induced by Rifampin (Chen et al., 2021). By inhibiting ER stress and preventing the ubiquitination degradation of Multidrug resistance-associated protein 2, 4-PBA demonstrated the potential to reduce the concentration of serum total bilirubin. These findings suggest that 4-PBA might hold promise as a treatment option for managing Rifampin-induced cholestatic liver injury in the context of tuberculosis therapy. Various herbal components like pentacyclic triterpene celastrol reprogrammed the liver’s metabolism by either reducing the process of lipogenesis or promoting lipolysis (Fan et al., 2022). Nonetheless, the practical use of it in a medical context faces significant constraints due to its notably low bioavailability and prolonged gastrointestinal retention. Therefore, nanoformulations of pentacyclic triterpene celastrol were introduced with higher efficacy and enhanced bioavailability. The application of galactose-coating has shown promise in improving the targeted delivery of different types of nanoparticles, such as nanomicelles made of PEG-PCL, nanocrystalline particles, and albumin nanoparticles, for the purposes of therapy and radiolabeled diagnosis (D'Souza & Devarajan, 2015). Likewise, the utilization of galactosylated nanoparticles has not only addressed the issue of bioavailability for numerous hydrophobic drugs but has also facilitated their liver-targeted delivery, including substances like curcumin. Consequently, there is an ongoing demand for the development of novel delivery systems that can further enhance biocompatibility and liver-specific delivery of drugs.

## 5. Small molecule-driven receptor-mediated ER targeting

The inositol 1,4,5-trisphosphate receptor (IP_3_R) and ryanodine receptors (RyR) stand out as the most widely distributed Ca^2+^-release channel within the ER (Woll & Van Petegem, 2022). The IP_3_R, along with the ensuing calcium release triggered by inositol 1,4,5-trisphosphate (IP_3_) activation (referred to as IP_3_-induced Ca^2+^ release or IICR), plays a pivotal role in a multitude of cellular processes, including the intricate regulation of cell destiny. Elevated intracellular Ca^2+^ levels initiate the activation of RyR, which subsequently depletes Ca^2+^ within ER, leading to a further increase in cytosolic Ca^2+^. During ischemia, energy reserves are depleted, neurons undergo depolarization, and ionic balance is disrupted, activating glutamate receptors (GluR), Ca^2+^ channels, and the concurrent release of excitotoxic glutamate (Su & Li, 2016). In recent decades, sigma receptors (SRs), encompassing subtypes sigma 1 (S1R) and sigma 2 (S2R), have gained substantial attention in relation to conditions associated with aging and mitochondria, including but not limited to Parkinson’s and Alzheimer’s disease, multiple sclerosis, and amyotrophic lateral sclerosis. TMB-8 (8-(N,N-diethylamino)-octyl-3,4,5-trimethoxybenzoate), a blocker of Ca^2+^-induced Ca^2+^ release, was employed following insults mediated by α-amino-3-hydroxyl-5-methyl-4-isoxazole-propionate (AMPA) receptors as reported by Ruiz et al. (Luo et al., 2012). This intervention effectively mitigated oligodendrocyte cell death and the excessive accumulation of cytosolic Ca2+. Concurrently, inhibiting RyR reduced the burden of Ca^2+^ overload, whereas inhibiting IP3R proved ineffective. Moreover, AMPA-triggered events, such as mitochondrial membrane depolarization, oxidative stress, and caspase-3 activation, were consistently diminished when RyR inhibition was applied. Additionally, AMPA stimulation induced an ER stress response, evident through phosphorylation of the α subunit of eukaryotic initiation factor 2α, overexpression of GRP chaperones, and RyR-dependent cleavage of caspase-12. Importantly, alleviating ER stress with salubrinal offered protection to oligodendrocytes against AMPA-induced excitotoxicity (Luo et al., 2012). These findings collectively demonstrate that RyR-mediated Ca^2+^ release plays a pivotal role in cytosolic Ca^2+^ overload, mitochondrial dysfunction, ER stress, and cell death resulting from AMPA receptor-mediated excitotoxicity in oligodendrocytes.

The efflux of Ca^2+^ is facilitated by chloride channels located in the membrane of the ER, as confirmed through several electrophysiological investigations. These investigations have provided substantial evidence supporting the notion that chloride channels in the ER membrane serve as a counterion pathway, contributing to the process of Ca^2+^ efflux (Lee et al., 2020). Additionally, the binding capacity of chloride with the chloride pump in the ER has been explored, offering the potential for ER-targeted strategies. In pursuit of these objectives, scientists have incorporated a chlorine group as a component for targeting the ER. They have successfully developed a two-photon fluorescence probe named TPFL-ER, which is derived from fluorene. This probe utilizes the chlorine group to facilitate its specific localization within the ER, allowing for effective two-photon fluorescence detection within this cellular compartment (Zhang et al., 2013). This probe has demonstrated superior performance compared to commercially available ER-trackers, exhibiting enhanced photostability, reduced background signal, lower toxicity, and improved accumulation within the ER. A separate research study demonstrated the successful creation of a remarkably effective fluorescence detection probe for biothiols, utilizing BODIPY. Within this probe, the chlorine group is integrated, allowing for specific binding with the chloride pump found in the ER. As a result, this probe achieves precise localization within the ER, enabling real-time detection of biothiols within this cellular compartment (Tang et al., 2022).

The compounds with sulfonyl groups exhibit a notable preference for the ER due to their specific interaction with the ATP-sensitive K + channel, known as sulfonylurea receptors, which are prominent on the ER membrane through cyclohexyl sulfonylurea fraction. The derivatives of glibenclamide are considered fundamental sulfonyl ligands, and they are commonly used commercial fluorescent probes for ER-based live cell imaging, namely ER-Tracker Red (BODIPY™ TR-glibenclamide) and ER-tracker Green (BODIPY™ FL-glibenclamide), consist of both BODIPY and glibenclamide (Wijesooriya et al., 2019). While the glibenclamide-based ER targeting system exhibits lower cellular toxicity, the comparatively large molecular weight (MW) and exclusive pharmacokinetics and pharmacodynamics properties of glibenclamide may have unintended effects on the normal biological functions of the ER (Vetere et al., 2022). Thus it is recommended to use these ER-trackers at the lowest possible concentrations. Moreover, the high cost of these tracking probes poses further challenges to their widespread applications. Given these factors, there is an urgent need to develop novel ER-targeting optical probes or trackers that are characterized by lower MW and cost-effectiveness. The sulfonamide fraction, known for its immense affinity and distinct selectivity toward the sulfonamide receptors abundant in ER, has been extensively utilized in receptor-mediated ER active targeting strategies (Masoudifar et al., 2022). Among the well-known sulfonamide compounds, p-toluenesulfonamide or 4-methylbenzenesulfonamide is incorporated into the commercially available ER-tracker dye Blue-White DPX (Dapoxyl), enabling notable localization with the ER. Building upon this foundation, the development of ER-targetable fluorogenic probes, leveraging the physicochemical properties of the ER microenvironment, has been on the rise to cater to various applications. One promising example is the CNSB (cyano-substituted stilbene) fluorescent probe, which operates on the principle of FRET (Forster resonance energy transfer). It comprises a blue fluorescent coumarin and red fluorescent probe 4-amino-7-aminosulfonyl benzodiazole to monitor the transition of metal ions, particularly copper, which plays a crucial role in biological signal transmission (Shi et al., 2021).

The introduction of the sulfonate group in the amphoteric ionic probe, known as zwitterion type NIR AIEgens, confers specific ER-targeting capabilities (Hussain et al., 2018; Zhu et al., 2021). This targeting ability is likely attributed to the electrostatic force of attraction between the amphoteric molecule and the cationic phosphocholine cytidylyltransferase present on the negatively charged surface of the ER membrane (Cornell & Ridgway, 2015). The incorporation of the sulfonate group enables selective interaction with the ER, allowing for efficient localization and potential applications of these zwitterionic AIEgens in ER-related studies. In contrast, when the sulfonate group is replaced with p-bromobenzene, it can lead to a mitochondria-specific targeting event. This suggests that the amphiphilic property is a crucial prerequisite for achieving specific accumulation in the ER (Cerrato et al., 2017). This modified probe, characterized by better in vitro stability in the presence of light, lower photobleaching, and improved cell viability, along with its higher tracking efficiency, emerges as a promising optical probe for explicit single-photon and two-photon excited fluorescence imaging of the ER in living biological cells. Its selective targeting capability and favorable properties make it a valuable tool for studying ER dynamics and processes in a cellular context.

### 5.1. Flavonoids targeting ER stress

Flavonoids have garnered considerable interest due to their diverse pharmacological effects, spanning antimicrobial, anti-inflammatory, and cancer-preventive effects (Deepika et al., 2023; Lazer et al., 2018). Two phenyl functions at positions 3 & 8 distinguish morusin from other flavonoids found in *Morus australis*. A recent study examined the effects of morusin on human epithelial ovarian cancer. The findings revealed that morusin had the ability to trigger ER stress, leading to an increase in the expression of several proteins, including BiP, CHOP, IRE1α, and p-eIF2α (α-subunit of phosphorylated eukaryotic initiation factor-2) (Martucciello et al., 2020). Furthermore, the treatment resulted in a cell death process resembling paraptosis, which was characterized by the enlargement of the ER and mitochondria. This phenomenon was attributed to the release of calcium ions from the ER into the mitochondria. Bavachin is another natural flavonoid that inhibits cell proliferation, mitochondrial apoptosis, and ER stress reducing the level of Mfn2 protein (Yang et al., 2023). The -OH group at the 5-position of 5-hydroxy-7-methoxyflavone plays a crucial role against uncontrolled tumor cell proliferation after binding to the cell membrane to enhance the cellular uptake leading to ROS generation and calcium release. This causes ER stress and alters the mitochondrial membrane potential. Furthermore, the increasing concentration in the cytosol reduces the Bcl2/Bax facilitating Caspase-3-dependent apoptosis advancement (Kavya et al., 2013). Phenylate coumarin, auraptene, induces apoptosis by ER stress that activates caspase-8 & 12 and c-Jun N-terminal kinase (JNK). Natural polyphenol curcumin is also responsible for ER stress-driven apoptosis in several cancer cells, increasing the intracellular calcium content that inhibits ATPase pump and activates the signaling of Ca^2+^/calmodulin-dependant protein kinase-2 (CaMK2), leading to apoptosis and dysfunction in mitochondria (Zhang et al., 2018).

### 5.2. ER targeting connected with protein translation

Protein synthesis plays a crucial role in the development and spread of cancer, contributing to the uncontrolled growth, fast multiplication, and circulation of cancerous cells. Using mRNA translation targeting to eliminate cancer cells selectively has emerged as a promising strategy in recent years (Ortega et al., 2021). The initiation, elongation, termination, and recycling of ribosomes are all involved in mRNA translation in eukaryotes. Initiation, particularly, holds significant importance in translation regulation and is often disrupted in cancer due to alterations in the expression and phosphorylation of key initiation proteins such as eIF2α, eIF3, and eIF4F. Oncogenic signaling pathways like MAPK (mitogen-activated protein kinase) and PI3K-AKT-mTOR (Phosphatidyl-inositol-3-kinases/serine-threonine kinases/mammalian target of rapamycin) influence translational control, which, in turn, maintains several oncogenic programs (Truitt & Ruggero, 2016). Activation of mTOR through the PI3K signaling cascade leads to the assembly of the eIF4F complex, enabling cap-dependent translation in response to energy and nutrient demands (Ma & Blenis, 2009; Holz et al., 2005). Consequently, components and regulators of the translation machinery become potential therapeutic targets for selectively combating cancer cells. Among these targets, harmine, a natural β-carboline, has demonstrated anticancer properties by inhibiting DYRK1A (dual-specificity tyrosine phosphorylation-regulated kinase 1 A), a protein kinase associated with tumorigenesis, and through its DNA intercalating properties (Hu et al., 2021). While harmine exhibits growth inhibition, 2,7,9-tri-substituted β-carbolines, although not affecting DYRK1A, have shown potential as protein synthesis inhibitors (Carvalho et al., 2017). By optimizing their pharmacological and physicochemical properties, the lead compound CM16 has been identified as a protein synthesis inhibitor in cancer cells.

## 6. ER targeting via lipophilic membrane

Biomembranes consist predominantly of lipids and proteins, exhibiting inherent amphiphilic properties. DiOC6 (3,3′-dihexyloxacarbocyanine iodide) is a readily available fluorescent sensor commonly used to visualize the ER in live cells (Colston et al., 2003). This lipophilic cationic compound demonstrates an ability to effectively distribute itself within the cytoplasmic membrane or the membranes of different subcellular organelles. Such distribution is achieved through electrostatic interactions. Nevertheless, the distribution of DiOC6 lacks selectivity due to its concentration-dependent membrane preference. Specifically, at low concentrations, DiOC6 predominantly localizes within the mitochondria, while at higher concentrations, it exhibits specific accumulation in the ER (McGill et al., 2005). This concentration-dependent shift in distribution allows for targeted visualization of different cellular compartments using DiOC6. Hydrophilicity and lipophilicity should be taken into account when designing hydrophobic ER probes. To address the challenges associated with the solubility and nonselective distribution of BF2-chelated BODIPY dyes in biomembranes, modifications can be made by incorporating cyclic oligoethylene glycol parts or acyclic form of the same (Kamkaew et al., 2015). These modifications increase the hydrophilicity of the dyes. As a result, two probes with improved hydrophilic nature are gained. When employing NIR radiation, these modified probes demonstrate a distinct distribution pattern in comparison to the original dyes. Specifically, they accumulate to a lesser extent in the plasma membrane but show increased accumulation in the ER. This shift in distribution allows for more specific and targeted visualization of the ER using these hydrophilic probes when excited with NIR light. In ER stress-dependent PDT, phosphorus oxide accumulates within neutral lipid structures, specifically in ER, and is used as a water-repellent or hydrophobic, classic organic type I PS (photosensitiser) (Zhuang et al., 2020). By capitalizing on the advantageous properties of phosphorus oxide, including its high stability, electrophilic nature, lower cytotoxicity, and membrane permeability, researchers introduced trianiline and pyridine moieties to enhance its functionality. As a result, two isomers were obtained, namely α-TPA-PIO and β-TPA-PIO. These isomers exhibit excellent aggregation-induced emission (AIE) properties, meaning they emit light more efficiently when they aggregate (Ni et al., 2021; Li et al., 2022). The incorporation of trianiline and pyridine moieties not only enhances the optical properties of phosphorus oxide but also expands its potential applications in ER targeting. Additionally, fluorescent probes specific to the ER can be designed using fluorinated hydrophobic Rhodol. Also, 5-nitrofuran-2-acrolein, acting as an electrophilic ligand, can combine with the protein p97 complex present on the membrane of ER (Shi et al., 2021). This interaction leads to a higher selectivity for the ER, inhibiting p97 and inducing responses based on misfolded protein, which ultimately contribute to the apoptotic death of cancer cells. Due to the favorable lipophilic nature and higher membrane potential of the ER membrane, metal complexes possessing hydrophobic properties and a relatively positive charge demonstrate a certain degree of ER targeting ability.

### 6.1. Metal nanoparticles in ER stress

It is crucial to have a thorough understanding of the physicochemical properties of nanomaterials and their interactions with biological systems in the present era. The biomedical applications of nanoparticles are influenced by their compositions, size, surface properties, and shape, and these properties determine the transportation of nanomaterials in the biological system, their biological kinetics, and their involvement in biomolecular signaling (Ghosh et al., 2019; Deepika et al., 2018; Haribabu et al., 2021). There have been reports that engineered metal nanoparticles like Au, Ag, Cu, etc., along with several metal oxide nanoparticles, induce cytotoxicity that can be detected by the generation of ROS (Pallavi et al., 2023). However, different nanomaterials’ toxic assessments have not clearly outlined the correlation between ROS production and ER stress response. As an example, silver nanoparticles (AgNPs) modulate ER stress to induce apoptosis (Quan et al., 2020). Furthermore, recent studies have found that several nanoparticles can induce ER stress by triggering different cellular response dependant pathways, including apoptosis or other inflammations. There is an important role of oxidative stress in most of the mechanisms that contribute to the ecotoxicological effects of nanomaterials through the generation of ROS by the chemically active nanoparticle surface and compositions (Buchman et al., 2019). In addition, some nanoparticles cause inflammation and interfere with the immune system directly. There is also evidence to suggest that nanoparticles alter the functions of lysosomes and cause autophagy in the cells. Nanoparticles’ ability to induce ER stress has recently been recognized as a promising mechanism for inducing cellular toxicity in recent years based on a deeper look at their mechanism of action.

### 6.2. Gold nanoparticles (AuNPs) and AgNPs

High atomic number elements, such as gold, iodine, or iodine-based nucleoside analogs like iodo-deoxyuridine, are favorable radiosensitizers due to enhancing the biological effects of X-ray radiation (Girigoswami et al., 2009). Studies have shown that AuNPs with a size less than 200 nm act as favorable radiosensitizers for tumor cells or free cell suspensions, but AuNPs with a diameter lesser than 6 nm are efficient in targeting specific cell organelles (Kodiha et al., 2015). The stability, biocompatibility, and bioavailability of AuNP for the longer circulation time can be controlled by modifying the surface with various water-soluble nontoxic polymers like polyethylene glycol (PEG). The non-immunogenicity of the polymers helps in the reduction of the RES uptake also. The production of ROS and apoptosis of AuNPs as well as the regulation of the cell cycle, have also been observed to be different biological effects of AuNPs. Noel et al. report that AuNPs did not increase the expression of cell surface proteins like vimentin etc., while treated but instead promoted their degradation (Noël et al., 2016). They demonstrated that AuNPs activate ER stress as a result of Caspase-4 processing by activating IRE1, ATF6, and PERK. A variety of cell organelles and compartments are connected to endosomes or lysosomes through which many AuNPs enter the cells. According to the study of Samhadaneh et al., gold nanourchins have been found to activate the PERK expression of UPR (Samhadaneh et al., 2019). Additionally, they promote the oxidation of RNA, facilitate the formation of P-bodies and lead to the accumulation of the oxidative stress markers Nrf2 (nuclear factor erythroid 2-related factor 2) and NFkB (nuclear factor-kappa B) in nuclei. Collectively, these findings suggest that gold nanourchins disrupt homeostasis in various cellular processes, including ER function, redox balance, protein expression regulations, and RNA stability.

The applications of AgNPs have been extensively utilized to promote RSO-dependant oxidative stress and cytotoxicity. The exposure of cells to AgNPs results in membrane leakage, impaired mitochondrial functions, and reduced viability across various cell types. These effects highlight the potential adverse impacts of AgNPs on cellular health and functionality. The cytotoxicity induced by AgNPs is closely associated with oxidative stress, and emerging evidence suggests that the ER plays a significant role in AgNPs-mediated apoptosis. The important factors in ER stress, such as PERK proteins, IRE1, and ATF6, have been implicated in this process. ATF6, along with the spliced form of XBP1, positively regulates the expression of plenty of genes involved in ER stress, including GRP78. Induced apoptosis by AgNPs is associated with calcium overload in mitochondria, indicating that calcium homeostasis plays a key role. Quan et al. examined cellular and molecular mechanisms of AgNPs-mediated anticancer activity in HCT116 (colorectal cancer cells) cells and xenografted mice (Quan et al., 2021). AgNPs have been found to trigger mitochondrial ROS species production, leading to the dysfunction of mitochondria and activation of ER stress responses, specifically through the involvement of NOX4. These mechanisms contribute to the antitumor effects of AgNPs against colorectal cancer, primarily through the induction of apoptosis via pathways associated with ROS and ER stress-related mitochondrial processes. AgNPs were examined in human retinal pigment epithelium ARPE-19 cells to determine their ability to induce ER stress which was demonstrated by Quan et al. (Quan et al., 2020). The study revealed that AgNPs caused significant cytotoxicity in ARPE-19 cells and induced apoptosis through various cellular mechanisms. AgNPs dose-dependently triggered caspase-3 and PARP cleavage, mitochondrial membrane potential depolarization, and upregulated ER stress markers such as CHOP, PERK, eIF2α, IRE1, and ATF6. This indicates that AgNPs induce apoptosis in human RPE ARPE-19 cells through the activation of the ER stress response.

### 6.3. Metal oxide nanoparticles

A number of novel biomedical applications have been investigated for metal oxide nanoparticles, e.g., superparamagnetic iron oxide nanoparticles (SPION). In addition to contrasting ability in MRI, the SPION is also widely engaged in magnetic transfection, cancer cell separation or cell tracking, delivery of the drug to the diseased tissues or cells, hyperthermia to kill cancer cells, tissue repair, etc (Amsaveni et al., 2013). It has also been found that metal oxide nanoparticles have an excellent capacity to treat fungi, bacteria, and cancerous cells as well using ROS generation, most probably keeping health cells unaffected due to their outstanding biocompatibility. Ultrasmall paramagnetic iron oxide nanoparticles impact inflammatory signaling pathways, including the release of IL-6 from the infected cells (Saengruengrit et al., 2018). Studies showed that hepatocytes are exposed to ultrasmall iron oxide nanoparticles, which trigger the expansion of ER, upregulation of calcium ions, and induction of ER stress. The UPR and ATF4/PERK pathways are involved in IL-6-associated inflammation that SPION regulates. It is possible to reduce hepatic inflammation caused by SPION by targeting ATF4/PERK pathways. As a result of Mn3O4 nanoparticles, Yi et al. reported HAC1 mRNA splicing was observed in saccharomyces, and UPR genes were significantly upregulated, suggesting the induction of ER stress (Yi et al., 2017). The extracellular invertase activity as well as the surface ferric reductase activity, were greatly hampered during treatment, which might be due to interference of the secretion pathway caused by ER stress. Based on the results of Guo et al., silica nanoparticles increased lipid accumulation, cytotoxicity, and apoptosis when oxLDL is stimulated (Guo et al., 2023). A quantitative analysis of cholesterol influx and efflux genes revealed that CD36, along with SRA expression, were significantly upregulated, while ATP binding cassette expressions were significantly downregulated. ER is specialized for producing, modifying, and even trafficking lipids. Silica nanoparticles increased lipid accumulation by controlling cholesterol influx and efflux in macrophage cells, and oxLDL treatment triggered ER response, while silica nanoparticles exposure increased ER stress. As a result, silica nanoparticles may be responsible for triggering ER stress signaling and the foamy appearance of macrophages and apoptosis in response to oxLDL. Similarly, titanium dioxide nanoparticles elicit ER stress in human bronchial epithelium through the activation of IRE-1α phosphorylation (van Vliet et al., 2014). This activation leads to an increase in the expression levels of CHOP and GRP78/Bip. Consequently, the disruption of calcium homeostasis occurs. Since calcium levels are regulated by both mitochondria and the ER, there is a translocation of Ca2+ from the ER to mitochondria facilitated by mitochondria-associated ER membranes.

ER, stress has emerged as a promising target in cancer therapy, as it is closely associated with cancer hallmarks. However, effectively targeting the ER in a cancer cell environment remains challenging. In a recent study by Pandey et al., they addressed this issue by engineering self-assembled 3D spherical nanoparticles using graphene oxide (GO) that specifically targets the ER (Pandey et al., 2020). These ER-targeted GO nanoparticles were designed to contain dual inducers of ER stress, namely doxorubicin and cisplatin. The results of the study demonstrated that the ER-GO nanoparticles induced apoptosis mediated by ER stress in HeLa cells. Interestingly, the nanoparticles also activated autophagy, which could be inhibited by combination treatment with ER-GO nanoparticles and chloroquine. Furthermore, the nanoparticles showed efficacy in inducing ER stress-associated apoptosis in breast, lung, and drug-resistant triple-negative breast cancer cell lines (Pandey et al., 2020). The researchers envision that these self-assembled graphene oxide nanoparticles, specifically targeting the ER, can serve as a platform to exploit ER stress and the associated unfolded protein response (UPR) as a therapeutic target, leading to promising outcomes in cancer therapy. A novel nanoparticle was developed by combining functional GO with PEG, along with folic acid (FA) for targeting and indocyanine green (ICG) dyes as photosensitizers. This nanoparticle was designed to deliver a combination of an MTH1 inhibitor and doxorubicin (Huang et al., 2020). Upon testing, the PEG-GO-FA-ICG nanoparticle demonstrated the ability to effectively inhibit the proliferation and migration of osteosarcoma cells through a combined approach of chemo-photodynamic therapy. The enhanced chemo-photodynamic therapy exhibited dual effects by inducing both apoptosis and autophagy. This was achieved by suppressing the MTH1 protein and promoting the accumulation of ROS. Huang et al., in their study, revealed that autophagy acted as a protective mechanism against cell death, and inhibiting autophagy enhanced the anticancer effects of chemo-photodynamic therapy (Huang et al., 2020). However, it was observed that chemo-photodynamic therapy-induced apoptosis was associated with the occurrence of ER stress. It was proposed that ROS might contribute to ER stress, leading to apoptosis through the activation of the JNK/p53/p21 pathway. Treatment with palladium nanoparticles was found to have a positive impact on the biogenesis and release of exosomes. This effect was achieved by inducing oxidative stress, ER stress, immunomodulation, and apoptosis (Gurunathan et al., 2021). Notably, when THP-1 cells were pretreated with N-acetylcysteine, there were significant reductions in palladium nanoparticles-induced exosome biogenesis and release. These studies indicate that targeting oxidative stress and inhibiting specific pathways can effectively modulate the production of exosomes. Such an approach holds promise for improving the generation of exosomes for diverse therapeutic applications.

### 6.4. Lipid nanoparticle-mediated ER targeting

**Figure 6.**
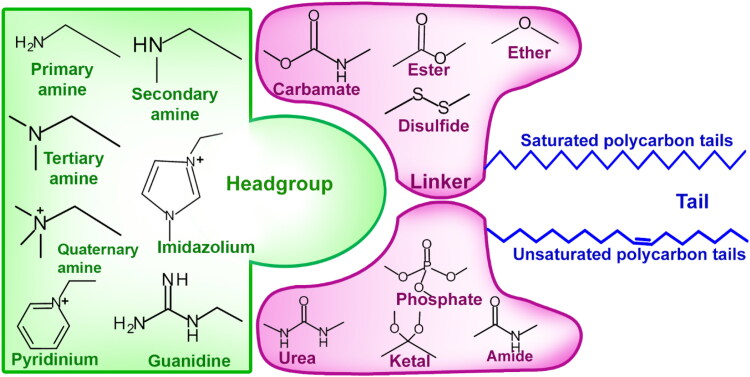
Amphiphilic structure of lipid molecules to formulate nanoparticles.

A lipid is an amphiphilic molecule with a polar head group, a polycarbon hydrophobic tail, and a linker molecule existing in between them (Figure 6). The lipid nanoparticles consist of a homogeneous lipid core, and nanostructured lipid carriers (NLC) differ from solid-lipid nanoparticles (SLN) in their matrix compositions (Sakellari et al., 2021). The solid lipid particle matrix is solid both at room and body temperature when it is substituted for the liquid lipid or oil in an oil in water microemulsions. Electrostatic interactions initiate self-assembly in cationic lipids and ionizable lipids (Rissanou et al., 2020). The delivery of nucleic acids continues to be carried out by lipoplexes containing cationic lipids. The use of pH-sensitive ionizable lipids has been recommended due to toxicity issues and the ineffective in vivo efficacy of cationic lipids. A lipid formulation is designed to produce lipid molecules that would show a net neutral charge in biological pH but exhibits a cationic charge within acidic endosomes once they are formulated into LNPs (Cárdenas et al., 2023). Ionizable lipids generally have a cationic head group with multiple numbers, but a single unit of the head group is very common. The amines, heterocyclic functional groups like pyridinium, imidazolium, etc., and guanidine are the common head groups present in the ionizable lipid molecules (Seo et al., 2023). The linker molecules join the head groups with the hydrophobic tails to determine the stability, bioavailability, cytotoxicity, biodegradability, and payload holding capacity. The common linkers include amide, carbamate, disulfide, ester, ether, ketal, phosphate, etc. The polycarbon hydrophobic tails play an important role in the formation of nanostructures and their efficiency (Viegas et al., 2022). The long tail groups determine the fluidity, dissociative nature in acid/basic medium, and lipophilicity, including fusion probability. An ionizable lipid molecule specifically holds 1 to 4 such saturated or unsaturated hydrophobic tails made of approximately eight to twenty carbon atoms.

Engineered lipid nanoparticles are very promising for their ability to carry mRNA and RNA-based vaccines due to their protective role against the digestion by RNases and neutralizing electrostatic interactions existing between RNA and the biological membranes. Similarly, lipid nanoparticles have recently been fabricated to target ER for inducing stress to facilitate apoptosis in tumor cells. Pandey et al. developed an oleic acid-mediated engineered lipophilic molecular assembly containing pan-Bcl2 inhibitor obatoclax for targeting ER and mitochondria in HeLa tumor cells (Pandey et al., 2020). They demonstrated that the lipid formulations enhanced internalization, facilitated ROS generation, and triggered apoptosis. Improved hydrophilicity of aza-BODIPY for intracellular imaging was achieved by fuzing two aromatic fragments with BF_2_-chelated azadipyrromethene dyes linked by oligoethylene glycol fragments, according to Kamkaew et al. (Kamkaew et al., 2015). Fluorescent molecules with oligoethylene glycol portions were found in the ER of fibroblasts, pancreatic cancer cells, and liver cancer cells, potentially making them useful for near-IR staining of ER. An emerging therapeutic strategy against neuronal degeneration was developed by Rokotoarisoa et al. that targets ER stress with multidrug-loaded cubosome and spongosome lipid nanoparticles (Rakotoarisoa et al., 2022). In the presence of these liquid crystalline nanoparticles made from monoolein, natural neurotrophic components such as curcumin, omega-3 fatty acids, and others were co-encapsulated. A cellular model of tunicamycin-induced ER stress was used to examine the neuroprotective properties of the nanoparticles using SH-SY5Y cells which are dedicated to human neuroblastoma study (Rakotoarisoa et al., 2022). Cell survival was enhanced by the brain-acquired neurotrophic component-loaded liquid crystal nanoparticles. The development of neuroprotective nanomedicine by exploiting ER stress-targeting mechanisms may be made possible by liquid crystalline nanoparticles with multi-drug loading.

### 6.5. Transportation of nanoparticles

**Figure 7.**
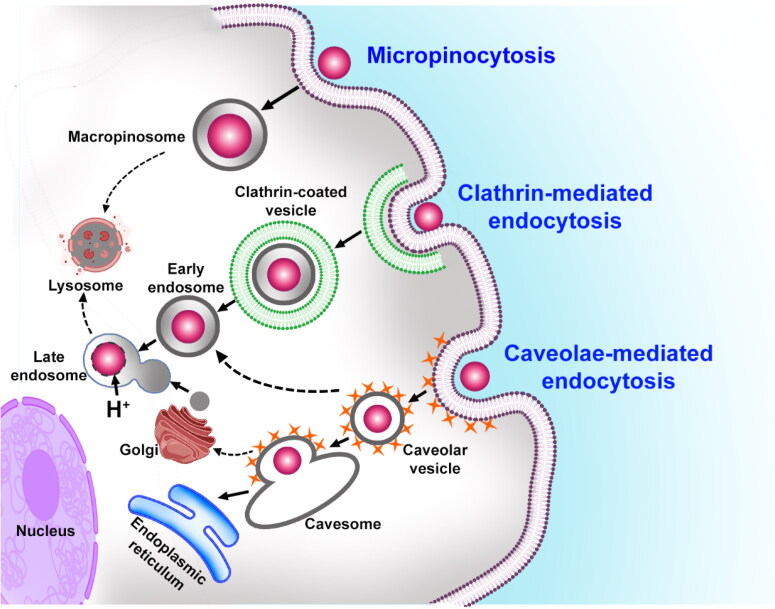
Schematic description of transportation of nanoparticles.

One proposed mechanism that plays a pivotal role in facilitating the entry of nanoparticles into tumors is referred to as the Enhanced Permeability and Retention (EPR) effect (Zhang et al., 2016; Mercy et al., 2023). This phenomenon involves two essential components: an elevated extravasation (enhanced permeability) of nanoparticles, attributed to the presence of a more permeable endothelial cell layer in tumor tissue, and a diminished drainage of nanoparticles by the lymphatic system (increased retention) within tumor sites (Vilella et al., 2015). The second mechanism at play is transcytosis, which is a dynamic metabolic process requiring endothelial cells to undergo cytoskeletal and cell membrane reorganization (Figure 7). This intricate process encompasses the formation of vesicles capable of engulfing nanoparticles, the development of diaphragms termed fenestrae, or the transportation of nanoparticles through the cytoplasm. In order for nanoparticles to transcytose across the tumor endothelium, they must initially undergo endocytosis within tumor endothelial cells. Several distinct pathways exist for endocytosis, although not all of them are necessarily conducive to transcytosis. The most prevalent among these pathways are clathrin-mediated endocytosis, caveolae-mediated endocytosis, and micropinocytosis (Sheth et al., 2021).

Clathrin-driven endocytosis represents a receptor-specific mechanism of internalization that employs vesicles coated with the triskelion protein known as clathrin. These vesicles are responsible for engulfing materials that bind to surface receptors. Clathrin itself does not directly interact with the cell membrane or its specific receptors; instead, it relies on several other proteins to facilitate binding and vesicle formation (Sheth et al., 2021). Caveolae-mediated process of endocytosis, on the other hand, constitutes another form of receptor-specific endocytosis centered around caveolae, membrane invaginations enriched with cholesterol and sphingolipids. Unlike clathrin, caveolin coats do not disassemble before merging with endosomes (Iversen et al., 2011). Nevertheless, their vesicles, similar to clathrin-mediated endocytosis, possess multiple destinations depending on their cargo. These endosomes may be directed toward lysosomes for degradation or transported to the Golgi bodies and endoplasmic reticulum to serve transcytotic functions. The initial phase in creating nanoparticles capable of effectively and selectively transcytosing through endothelial cells in tumor blood vessels involves precise targeting of endocytic pathways. A widely employed approach to achieve this entails surface modifications of the nanoparticles using molecular ligands tailored to interact with endocytic receptors. These modifications often include the attachment of essential intermediates such as transferrin, albumin, iRGD, EGF, and folic acid conjugates (Taiariol et al., 2022; Pallavi et al., 2022).

## Conclusion and viewpoints

In this review, we summarized the role of the endoplasmic reticulum and mitochondria in disease progression, including their pathophysiological significance and nanoenabled delivery systems to play a role in addressing the ER stress-related pathways. The pathogenesis of multiple diseases is closely related to the physiological peculiarity of the ER, particularly hyperactive ER stress. These include exclusively cancer, neurodegenerative disorder, liver disease, and diabetes mellitus. Tumor cells might also increase their tumorigenicity, metastasis, and resistance by exploiting their highly adaptive ER responses. There is also the possibility that when an ER load is excessive, malignant cells can also be programmed to die. A targeted control over ER, which has good controllability, great efficiency, and a wide range of application possibilities, may, therefore, be a breakthrough in detecting and treating ER-associated physiopathological conditions, as well as treating ER-associated diseases in general. The sole purpose of this review is to provide knowledge on the recent progress in the development of molecules and carriers, especially nanocarriers that are specifically designed or engineered to target ER. For example, the ER can be used as a cancer treatment target when it triggers ER stress-mediated cell apoptosis. However, nanomaterials may cause certain risks due to their potential toxicity. Due to this, most experiments are conducted in vitro on cells and tissues, and the consequences on organs and on the integrity of the body are known very less. A single organelle is often studied, but interaction among organelles is rarely considered. There is, therefore, a need to investigate multiple organelles in order to suppress tumors and observe apoptosis.

Although there have been some major advantages in nanomaterials and strategies for targeting the ER, there are still some challenges to overcome. In order to construct ER-targetable nanoparticles, it is still necessary to find ER-targeting molecules or ligands that can target the ER. It is usually difficult to achieve satisfactory ER-targeting results when using ER-targeting molecules as an endowment for nanoparticles, especially when their propensity for cytotoxicity is potential. The exact mechanism of ER targeting of ligand-free nanoparticles has not yet been clarified, but many have been developed up to now. There is a prevailing belief that the advancement of novel nanomedicines specifically designed for targeting the ER, along with therapeutic and imaging methods tailored to the ER, has the potential to enhance our ability to control cellular processes and outcomes associated with the ER. This progress is anticipated to lead to a more profound comprehension of biological activities linked to the ER. It is our hope that a greater array of strategies and nanoparticle-based interventions targeting the ER will soon enter the realms of preclinical and clinical development, bringing us closer to achieving precision in therapy at the subcellular level.

## Data Availability

All the data are available in the manuscript.
